# Semi-reciprocal polarization maintaining fibre coupler with distinctive transmission characteristics

**DOI:** 10.1038/srep17268

**Published:** 2015-11-27

**Authors:** Xinyue Wang, Freya Thomas, Ziyu Wang

**Affiliations:** 1 College of Information and Electrical Engineering, China Agricultural University, Beijing, China; 2 Department of Physics, Imperial College London, London, UK; 3 State Key Laboratory of Advanced Optical Communication Systems and Networks, Department of Electronics, Peking University, Beijing, China

## Abstract

Optical couplers are very important devices in optical communication systems and optical sensor systems. Several types of optical couplers with different materials or different transmission characteristics have been reported. Here we propose a semi-reciprocal polarization maintaining fibre coupler with unique transmission characteristics, which is distinct from conventional polarization maintaining fibre couplers and polarization beam splitters, and investigate the characteristics of the coupler theoretically and experimentally. The experimental results show that for circularly and elliptically polarized input light, the proposed coupler will act both as an in-line polariser and a conventional polarization maintaining fibre coupler. The output polarization extinction ratio of the transmission arm is 31.79 dB at a centre wavelength of 841 nm. For linearly polarized input light, the coupler will merely act as a conventional 3 dB polarization maintaining fibre coupler. The unique features of the proposed coupler enables the removal of polarisers from optical sensor systems and coherent optical communication systems, and reduces the insertion loss and production cost of the optical path. Therefore there is wide application for this device in optical sensor systems and optical communication systems.

Optical couplers are ubiquitous in optical communication systems, including wavelength division multiplexing systems, coherent optical communication systems, and optical networks^1^,^2^. Optical couplers are also important elements of optical sensor systems, such as optical gyroscopes, optical hydrophones, and optical current sensors, etc.^3^,^4^,^5^,^6^. To date, several types of optical couplers with different materials or different transmission characteristics^7^,^8^,^9^, such as waveguide couplers^10^, fused-taper single mode fibre couplers^11^, fused-taper polarization maintaining fibre (PMF) couplers, and photonic crystal fibre couplers have been reported^12^,^13^,^14^,^15^,^16^,^17^,^18^.

In this article, we propose a semi-reciprocal PMF coupler with unique transmission characteristics, which is distinct from both typical PMF couplers and polarization beam splitters (PBS), and analyse the transmission characteristics theoretically and experimentally. Conventional PMF couplers are reciprocal devices, which can combine or split the input lights and maintain their polarization states at the output ports^19^. The splitting ratio of the coupler is almost a constant when the input polarization states are changed, as shown in Fig. 1a. Other fused-taper fibre devices are PBSs. PBSs can split a circularly or elliptically polarized beam into two orthogonal linearly polarized beams, as shown in Fig. 1b, but cannot split a linearly polarized beam into two beams^20^.

Figure 1c,d show the transmission characteristics of the semi-reciprocal PMF coupler. When linearly polarized light is input, as shown in Fig. 1c, the proposed coupler will act as a typical PMF coupler. When elliptically or circularly polarized light is input, as shown in Fig. 1d, the transmission arm (port 1 ↔ port 3 and port 2 ↔ port 4) of the coupler will act as an in-line polariser, translating the elliptically or circularly polarized light into linearly polarized light with high polarization extinction ratio (PER), and the coupling arm (port 1 ↔ port 4 and port 2 ↔ port 3) will merely act as a typical PMF coupler. The unique features of the proposed PMF coupler enables the removal of the PMF polariser from optical sensor systems and coherent optical communication systems, and reduces the insertion loss and production cost of the optical path^21^. Therefore, this semi-reciprocal PMF coupler has wide application in optical sensor systems and optical communication systems.

## Results

### Theoretical basis

**Figure 2:**
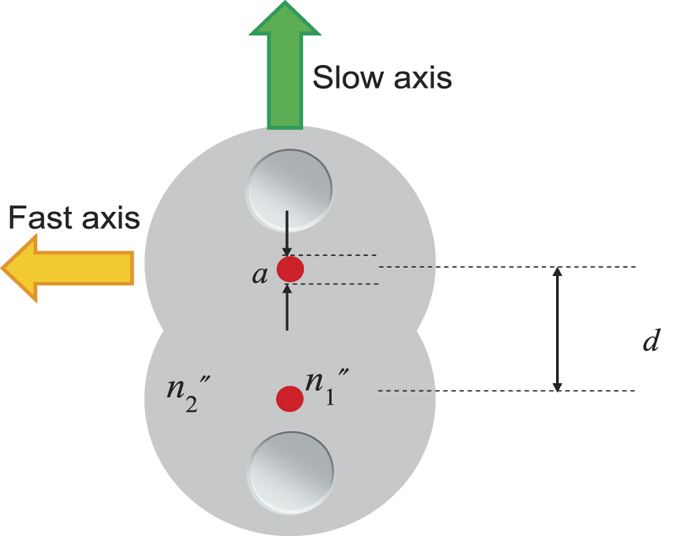
The cross-sectional schematic diagram of the semi-reciprocal PMF coupler.

Figure 2 shows a cross-sectional schematic diagram of the semi-reciprocal PMF coupler, where *a* is the radius of the fibre core, *d* is the distance between the centres of two cores at the fused-elongated section, and *n*_1_ and *n*_2_ are the core and cladding refractive index of the fibre, respectively.

In the approximation of weak coupling, the output intensity *P*_3_ at port 3, and the output intensity *P*_4_ at port 4 are given by:


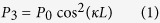



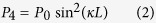


Where *P*_0_ is the power launched in port 1, *κ* is the coupling coefficient, and *L* is the coupling length. According to coupled-mode theory for optical fibres, the coupling coefficient of two identical and parallel step-index optical fibres can be expressed as:


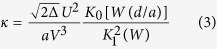


Where 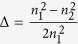, *K*_0_ and *K*_1_ are the modified Bessel functions of the second kind and of order 0 and 1, respectively. *V* is the normalized frequency of the core of an optical fibre, which is defined as:


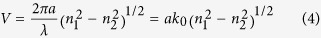


Where *λ* is the signal wavelength, and the parameters *U* and *V* satisfy:


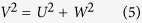



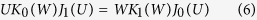


*J*_0_ and *J*_1_ are the 0-order and 1-order Bessel functions, respectively. Eq. (3) indicates that the coupling coefficient *κ* depends on the normalized frequency *V* and the normalized distance between the centres of two cores *d/a*.

The normalized frequency *V* should take a value that guarantees the single mode condition. For an optical fibre having a step type refractive index distribution, the single mode condition is guaranteed when *V*<=2.405 is satisfied. Eq. (4) indicates that the normalized frequency *V* depends on the parameters of the optical fibre. For polarization maintaining fibre, because of the mode birefringent index *n*_*b*_ = | *n*_1*x*_*– n*_1*y*_|, the normalized frequency *V*_*x*_ of *x* polarized light is different from the normalized frequency *V*_*y*_ of *y* polarized light. The coupling characteristics of *x* polarized light and *y* polarized light are not the same, and the coupling coefficient *κ* depends on the polarization of the light. The parameters of the PMF we used were as follows (http://www.fujikura.co.jp): wavelength λ = 850 *nm*, core radius *a* = 1.7 *μm*, core index *n*_1_ = 1.460, relative refractive-index difference between core and claddings (*n*_1_−*n*_2_)/*n*_1_ = 0.35%, mode birefringent index at used wavelength *n*_*b*_ = | *n*_1*x*_*– n*_1*y*_| = 5 × 10^−4^. Therefore, for the PM fibre we used, the values of normalized frequency were *V*_*x*_ = 1.5337 and *V*_*y*_ = 1.4566, respectively.

### Simulation and design

**Figure 3:**
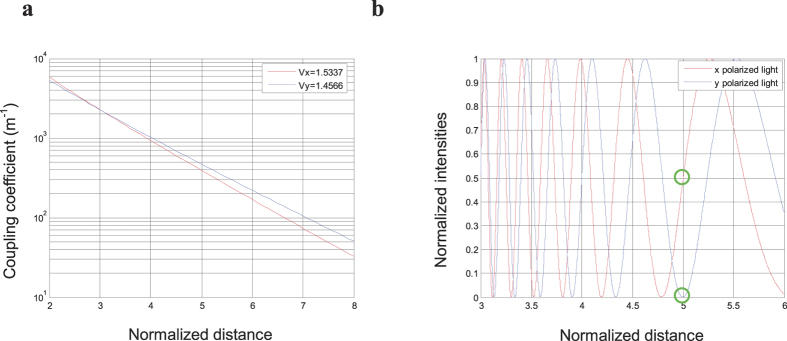
Examples of simulation results. (**a**) Coupling coefficients *κ*_*x*_ and *κ*_*y*_ of *x* polarized light and *y* polarized light as a function of the normalized distance between the centres of two cores *d/a*. The red line represents the coupling coefficient *κ*_*x*_ decreasing exponentially for *V*_*x*_ = 1.5337, and the blue line represents the coupling coefficient *κ*_*y*_ decreasing exponentially for *V*_*y*_ = 1.4566. The coupling coefficient *κ* is changed by varying the normalized distance *d/a* between the centres of two cores. (**b**) Normalized light intensities of *x* polarized light and *y* polarized light at port 3 of the coupler as a function of the normalized distance *d/a*. The red and blue lines represent the normalized light intensities of the x polarized light and y polarized light, respectively.

In Fig. 3a, the coupling coefficients *κ*_*x*_ and *κ*_*y*_ are plotted for *x* polarized light and *y* polarized light, respectively. When the centres of two cores are close to each other (normalized distance *d/a* is approximately 3 in Fig. 3a), *κ*_*x*_ ≈ *κ*_*y*_, so that the coupling characteristics of the *x* polarized light are almost the same as for *y* polarized light. The coupling coefficient *κ* depends slightly on the polarization of the light. This is the case for the conventional PMF coupler, which combines or splits the input lights and maintains their polarization states at the output ports. The splitting ratio of ports 3 and 4 is almost a constant when the input polarization states are changed.

When the distance between the centres of two cores is greater than a threshold, the difference between the coupling coefficients *κ*_*x*_ and *κ*_*y*_ becomes obvious. The coupling coefficient *κ* depends on the polarization of the light, therefore the coupling characteristic of *x* polarized light is totally different from *y* polarized light. For polarization maintaining optical fibres, Eq. (1) is satisfied for both the *x* polarized light and *y* polarized light. When the value of the coupling coefficient for *x* polarized light *κ*_*x*_ and *y* polarized light *κ*_*y*_ satisfies:


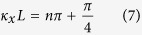



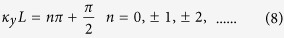


the output intensities of *x* polarized light and *y* polarized light at port 3 can be expressed as:


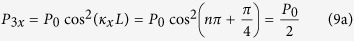



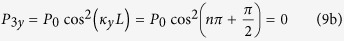


which is the case for the proposed semi-reciprocal PMF coupler.

Figure 3b shows the normalized light intensities of *x* polarized light and *y* polarized light at port 3 of the coupler calculated as a function of *d/a* by assuming that the coupling length *L* of the coupler is 10 *mm* in Eq. (9). When the normalized distance *d/a* between the centres of the two cores is approximately 5 as shown in Fig. 3b, almost all *y* polarized light passes through port 4, while little light output is measured at port 3. Conversely, the measured light intensity of *x* polarized light at port 3 is almost the same as at port 4. This demonstrates the special transmission characteristic of the semi-reciprocal PMF coupler. Therefore the normalized distance *d/a* between the centres of two cores can be designed to be 5, as shown in Fig. 3b.

The analysis therefore predicts that if *x* polarized light is applied to port 1, the coupler will act as a conventional 3 dB fused-taper PMF coupler. Conversely, if we apply circularly/elliptically polarized light to port 1, port 3 will output linearly polarized light with a high PER = 10log(*P*_*x*_/*P*_*y*_), and port 4 will output elliptically polarized light. This means that for circularly/elliptically polarized input light, the transmission arm will act as an in-line PMF polariser translating the circularly/elliptically polarized light into linearly polarized light with a high PER, and the coupling arm will act as a typical PMF coupler. The above discussion uses coupled-mode optical fibre theory to investigate the expected transmission characteristics of the proposed coupler. We now present the results of experimentation.

### Experimentation

**Figure 4:**
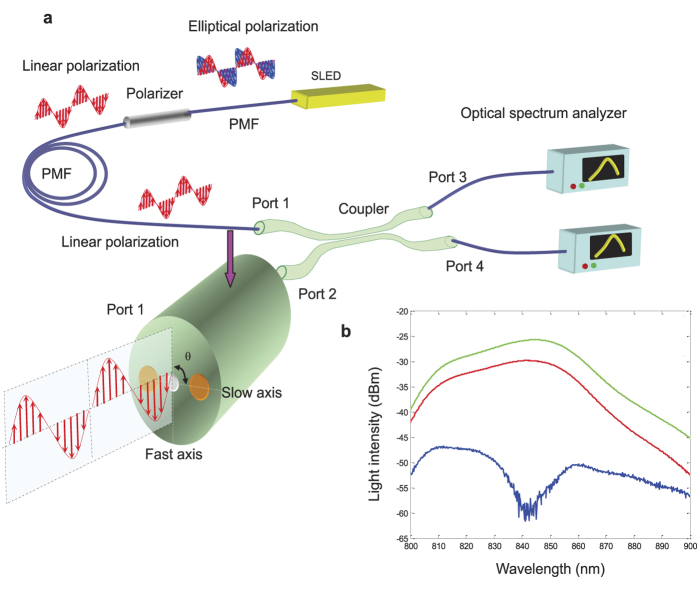
Experimental setup used to evaluate the semi-reciprocal PMF coupler. (**a**) Experimental setup. (**b**) The input light intensity at port 1 and the output light intensities at port 3 measured as a function of wavelength. The green line represents input light intensity at port 1, red and blue lines represent the output light intensities of *x* polarized light and *y* polarized light at port 3, respectively.

Figure 4a shows the experimental setup used to evaluate the proposed PMF coupler. Light from a broadband super luminescent light emitting diode (SLED, EXALOS-EXS210027-02, 3-dB centre wavelength 835.9 nm, 3-dB bandwidth 50.8 nm, 10-dB centre wavelength 838.1 nm, 10-dB bandwidth 76.3 nm, http://www.exalos.com) is polarized by a fibre optical polariser as shown in Fig. 4a. The linearly polarized light with PER of over 35 dB is launched into port 1 of the coupler. θ is the angle between the light’s polarization direction and the slow axis of port 1. Therefore, there are two orthogonally polarized light arrangements which can be applied: in one, *x* polarized light is input, corresponding to an input polarization angle of θ = 0°, and in the second, *y* polarized light is input, corresponding to input polarization angle of θ = 90°. The input light intensity at port 1, and the output light intensities at port 3, are measured using optical spectrum analysers (YOKGAWA AQ6370C) as shown in Fig. 4b. The wavelength range is from 800 nm to 900 nm. The output light intensity values of *x* polarized light and *y* polarized light are measured as a function of wavelength in steps of 0.2 nm.

## Discussion

**Figure 5:**
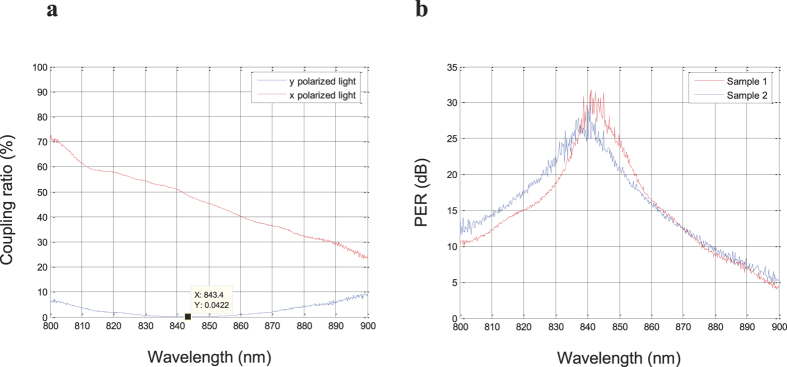
Experimental results of coupling ratio and output PER. (**a**) Output light intensities of *x* polarized light and *y* polarized light as a function of wavelength in steps of 0.2 nm. The red and blue lines represent the normalized light intensities of the *x* polarized light and *y* polarized light, respectively. (**b**) Relationship between the wavelength and the output PER of port 3 when applying circularly polarized light to port 1. The red line represents the output PER of sample 1, and the blue line represents the output PER of sample 2.

Figure 5a shows the relationship between the wavelength and the coupling ratio of the semi-reciprocal PMF coupler. The coupling ratio of the proposed coupler of *x* polarized light and *y* polarized light are plotted as a function of wavelength in steps of 0.2 nm. The coupling ratio of the 2 × 2 coupler is defined as *P*_3_/(*P*_3_ + *P*_4_), where *P*_3_ and *P*_4_ are the output light intensities at port 3 and port 4, respectively. Therefore, the coupling ratios of *x* polarized light and *y* polarized light are defined as *P*_3*i*_/(*P*_3*i*_ + *P*_4*i*_), (*i* = *x*, *y*), where *P*_3*x*_ and *P*_4*x*_ are the output light intensities at port 3 and port 4 when input light is *x* polarized, and *P*_3*y*_ and *P*_4*y*_ are the output light intensities at port 3 and port 4 when input light is y polarized.

When *x* polarized light (corresponding to an input polarization angle of θ = 0°) is applied to port 1 of the proposed coupler, the output light intensity of *x* polarized light at port 3 is almost the same as at port 4 at a wavelength of 843 nm, as shown in Fig. 5a. Therefore, the coupling ratio of *x* polarized light is *P*_3*x*_/(*P*_3*x*_ *+* *P*_4*x*_) = 52% at a centre wavelength of 843 nm. Conversely, when *y* polarized light (corresponding to an input polarization angle of θ = 90°) is applied to port 1, almost all the light intensity passes through port 4, while little output light intensity is measured at port 3 at wavelength 843 nm. Thus the coupling ratio of *y* polarized light is *P*_3*y*_/(*P*_3*y*_*+P*_4*y*_) = 0.042% at a centre wavelength of 843 nm, as shown in Fig. 5a.

Figure 5b shows the experimental results of output PER at port 3 if circularly polarized light is applied to port 1, measured as a function of wavelength. The wavelength range is from 800 nm to 900 nm, and the PER value is plotted in steps of 0.2 nm. The experimental results show that the maximum output PER at port 3 of sample 1 is 31.79 dB with a centre wavelength of 841.2 nm, and the maximum output PER at port 3 of sample 2 is 29.86 dB with a centre wavelength of 840.8 nm as designed. A PER of over 25 dB is obtained in a wavelength region of over 10 nm at approximately 840 nm, and a PER of over 20 dB is obtained in a wavelength region of 25 nm.

The experimental results prove that for *x* polarized input light, the proposed coupler has the function of splitting the input polarized light with a splitting ratio of port 3 to port 4 equal to about 52%:48%. On the other hand, for circularly and elliptically polarized light, the transmission arm (port 1 ↔ port 3 and port 2 ↔ port 4) of the proposed coupler has the function of converting the input light into linearly polarized light with a PER of 30 dB, while the coupling arm (port 1 ↔ port 4 and port 2 ↔ port 3) merely has a light splitting function.

In summary, we propose and demonstrate a semi-reciprocal PMF coupler with distinctive transmission characteristics, and investigate the coupler theoretically and experimentally. Experimental results show that for circularly and elliptically polarized input light, the semi-reciprocal coupler will act as an in-line PMF polariser and as a conventional PMF coupler, and the output PER of the transmission arm is over 30 dB with a centre wavelength of about 841 nm. For linearly polarized input light, the coupler will merely act as a conventional 3 dB PMF coupler with splitting ratio equal to about 52%:48%. The unique features of the proposed coupler enable us to remove the PMF polariser from optical fibre gyroscopes, optical fibre hydrophones, and coherent optical communication systems, etc. Therefore, this semi-reciprocal PMF coupler has wide application in optical sensor systems and optical communication systems.

## Methods

### Sample Fabrication

The proposed semi-reciprocal coupler has two PANDA fibres arranged side by side in such a way that their axes of polarization are parallel to each other as shown in Fig. 2. The fibres are heated and elongated in the longitudinal direction, thus forming a fused-taper section. The degree of separation of the two cores at the fused-taper section was adjusted to ensure the normalized distance *d/a* between the centres of the two cores was 5 by design. In the production of the fused-taper section, the temperature of the heating source was set lower than the temperature of a conventional PMF coupler, the distance from the heating source increased, and the elongation operation was performed quickly so as to reduce the amount of heat per unit time. Elongation was terminated when the coupling length *L* was 10 *mm* as desired, ensuring that the coupling ratio of *x* polarized light was about 50% and the coupling ratio of *y* polarized light was less than 0.05% with a centre wavelength of 840 nm.

## Additional Information

**How to cite this article**: Wang, X. *et al.* Semi-reciprocal polarization maintaining fibre coupler with distinctive transmission characteristics. *Sci. Rep.* **5**, 17268; doi: 10.1038/srep17268 (2015).

## Figures and Tables

**Figure 1:**
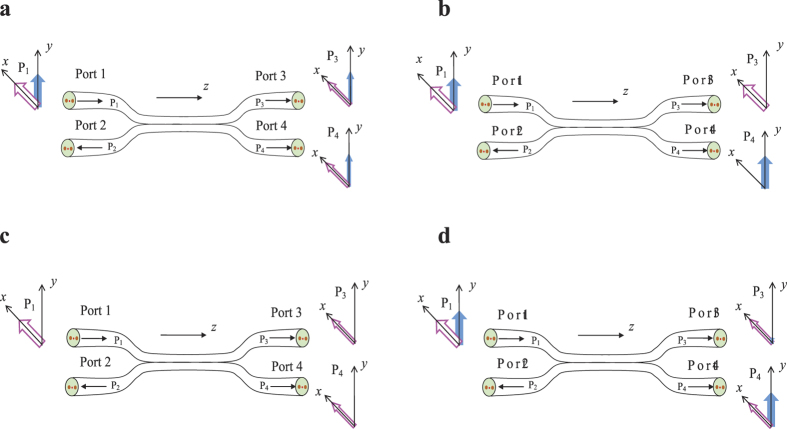
Transmission characteristics. (**a**) Transmission characteristics of a conventional PMF coupler. (**b**) Transmission characteristics of a PBS. (**c**) Transmission characteristics of a semi-reciprocal PMF coupler when linearly polarised light is input. (**d**) Transmission characteristics of the semi-reciprocal PMF coupler when elliptically and circularly polarised light is input. Pink and blue arrows represent the light intensities of the *x* and *y* polarized light, respectively.
